# Supplementary material for: Chromothripsis during telomere crisis is independent of NHEJ, and consistent with a replicative origin
Source: Genome Res. 2019 May;29(5):737–49. doi: 10.1101/gr.240705.118 (PMC6499312; doi:10.1101/gr.240705.118)
Supplement: Supplemental Material [file supp_gr.240705.118_Supplemental_file_1.zip › contigs/annotated_contigs/DB57/contig.3.DB57_length_570_mean_cov_5.84736842105.docx]

**DB57_length_570_mean_cov_5.84736842105**

```
CTCCTGGGTTCAAGCAATTCACCTGCCTCAGCCTCACAAGTAGCTGGGATTACAGGCACGTACCACCATGCCCGGCTAATTTTTGTATT
 >chr5:118140998-118141395 + E=2e-227
TTTACTAGAGATGGGGTTTCGCCATGTTGGTTGGCCAGGCTGGTCTCGAACTCCCAACCTCAGGCGATCCACCAGCCTCGGCCTCCCAA

AGTGCTGGGATTACACGCGTGAGCCATCACGCCCAGCAGTGGAAATTCTTAAAAATGCTTTTAGTTCCTTTGAATTAGTCATTCAATCT

CTCACACCTACAATTCTCCCCTACCCCAGATAAATACTTACCCCCTCGCTCAGTCCCTTCAAACCAACAACACTTCTTCCTCACTCCAC

TTTCTTTTTGGTCCTTTTCCATATCAAATGTGGCTGTTA|TT|TTTTATAAGGACAGCAATCCTATTCATGTGGCCTCCAACCATATGA
                                        >chr5:118343894-118344000 + E=4e-52 p=0e+00
CCTATCATCTCCAAAGGCCCCACCTCCCAACAACCATATGACCTATCATCTCCCAAAG|TACGCAAAGTGTATATT|ATATACTGTACA
                                                                            >chr8:43093
TAGAATTTCAAAGTACTCAAAGTATATATAATATACT|ATA101-43093150 - E=9e-03
```
